# Comparison of Superior Mesenteric Artery Remodeling and Clinical Outcomes between Conservative or Endovascular Treatment in Spontaneous Isolated Superior Mesenteric Artery Dissection

**DOI:** 10.3390/jcm11020465

**Published:** 2022-01-17

**Authors:** Sz-Han Yu, Ing-Heng Hii, I-Hui Wu

**Affiliations:** 1 Department of Surgery, Cardiovascular Section, National Taiwan University Hospital, Taipei 10002, Taiwan; a0915957593@gmail.com; 2 Department of Surgery, Cardiovascular Section, Dalin Tzu Chi Hospital, Chaiyi 62247, Taiwan; heng_1014@hotmail.com; 3 Graduate Institute of Clinical Medicine, College of Medicine, National Taiwan University, Taipei 10002, Taiwan

**Keywords:** dissection, mesenteric artery, treatment outcome, endovascular stent

## Abstract

The aim of this study was to analyze the superior mesenteric artery (SMA) remodeling after initial conservative or endovascular treatment with a standardized definition and midterm outcomes in patients with spontaneous isolated dissection of the superior mesenteric artery (SIDSMA). This retrospective study enrolled patients with SIDSMA from January 2007 to August 2019. All patients were treated initially with conservative treatment. If they failed the medical treatment, they were converted to interventional treatment. The morphological endpoint was determined by the standardized SMA remodeling, and the clinical endpoints were determined by the in-hospital mortality, hospital stay, and the bowel-related mid-term mortality. A total of 34 consecutive patients with SIDSMA were identified. Twenty-three (67.6%) and eleven (33.4%) patients underwent conservative and interventional treatments, respectively. Clinical features and morphologic changes on CTA were available in 25 (73.5%) patients during the median follow-up of 23.3 months. Standardized SMA remodeling was significantly (*p* < 0.05) better in patients undergoing endovascular stenting, especially in patients with Yun’s IIb classification. There was no mesenteric ischemia or SMA aneurysm during follow-up period. Patients with SIDSMA can be treated safely with initial conservative treatment. However, significant portions of patients will require endovascular intervention due to the persistent symptoms. Clinically endovascular stenting could be performed successfully, and SMA remodeling was satisfactory during the mid-term follow-up.

## 1. Introduction

Spontaneous isolated dissection of the superior mesenteric artery (SIDSMA) is rare but increasingly diagnosed because of the widespread availability of high-quality computed tomography angiography (CTA), especially in the lack of specific lab tests and clinical signs. The etiology of SIDSMA remains unclear. Hypertension, genetic variants, and the mechanical stress at the anterior wall of the SMA near the convex curvature have all been proposed [1]. Previous reports [2,3] have indicated that risk factors of SIDSMA included male, smoking, middle-aged, hypertension, and Asian population [1,2,3,4,5,6,7,8]. Clinical presentations can range from incidental CTA finding without symptoms to acute abdominal pain and can progress to superior mesenteric artery (SMA) occlusion and mesenteric ischemia. The CTA morphology alone could not predict the clinical course [3,9,10]. Initial treatment can always be managed conservatively with bowel rest, hydration, blood pressure control, and analgesia, with or without anticoagulation. In asymptomatic patients, conservative treatment is safe, and none of these patients required secondary intervention [11]. In symptomatic patients, the symptom relief rate ranges from 66.8% to 86.6% after one week [1,12,13]. However, 5.0–44.0% percent of patients still fail the medical treatment or require conversion to interventional therapy, predominately endovascular SMA stent, with a small percentage (1.0–3.0%) receiving open revascularization if progressive mesenteric ischemia led to bowel resection [9,14]. Given the dissected nature, one might also expect the aneurysmal degeneration of SMA in the following years, resulting in recurrent symptoms, aneurysm rupture, or bowel ischemia. In order to determine the optimal treatment, positive SMA remodeling is paramount. In the current literature, there was no standardized definition and quantitative comparison for the positive SMA remodeling based on the CTA morphological change over the follow-up between the interventional and medical treatments [5,14,15,16,17]. The use of antiplatelet or anticoagulation therapy to prevent SMA thrombosis and promote positive SMA remodeling is also controversial [9,11,16].

In this study, we investigated the risk factors of failure of initial medical therapy, the comparison of SMA remodeling between the intervention and conservative treatments by the CTA morphological change in the mid-term follow-up, and the clinical outcomes by reviewing patients with SIDSMA undergoing initial conservative treatment.

## 2. Materials and Methods

### 2.1. Study Patients

This retrospective study included 34 consecutive patients with SIDSMA at the National Taiwan University Hospital between January 2007 and August 2019. The follow-up data were truncated in March 2020. This study was approved by the Institutional Review Board of National Taiwan University Hospital (NTUH–202104075RINA). An informed consent was waived for this retrospective review.

**Figure 1:**
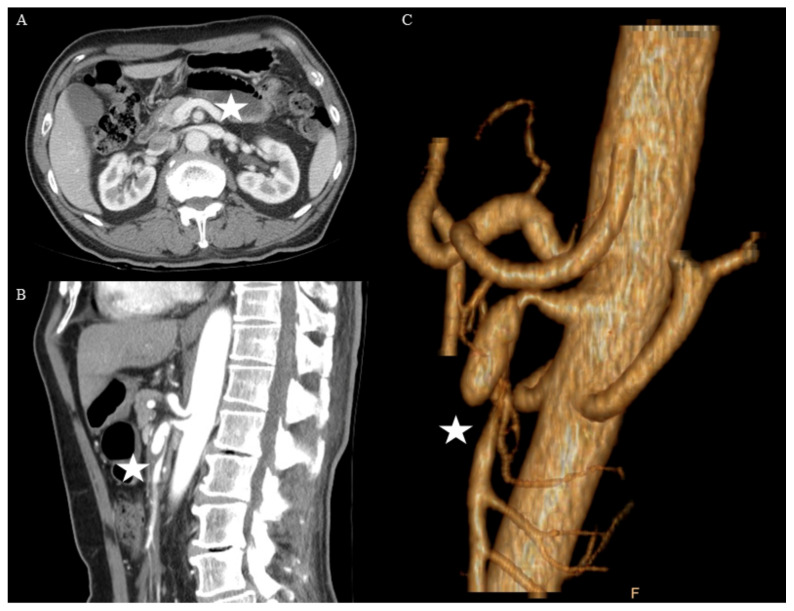
This was a 66-year-old man with Yun’s IIa spontaneous isolated dissection of the superior mesenteric artery (asterisks). Computed tomography (CT) scans, (**A**) axial, (**B**) sagittal, and (**C**) three-dimensional, demonstrated small patent true lumen, and conservative treatment was applied first.

The diagnosis of SIDSMA was made from findings on the axial views of CTA with an intimal flap in the SMA with/without false lumen (FL) thrombosis (Figure 1). Patients with concomitant aortic and SMA dissection were excluded from this study. Presenting symptoms included abdominal pain, nausea, and/or vomiting. The pain severity score was measured on a 10-point visual analog scale, and the duration of abdominal pain was also investigated. The morphological CTA findings adopted the Yun’s classification [3]. It classified the dissection of the SMA at CTA as type I (entry and re-entry tear visible, patent FL), type IIa (only entry tear visible, patent FL), type IIb (FL thrombosis, patent true lumen (TL), and type III (TL and FL occlusion). TL and FL diameters as well as the length of SMA dissection were recorded. Data collected on all patients included age, sex, comorbidities, medical histories, follow-up duration, length of hospital stay, change of symptoms, and treatment strategies. Morphology of the SMA dissection and morphological changes in the CTA follow-up were analyzed and compared between conservative and endovascular SMA stent (EVT) groups.

### 2.2. Treatment Algorithm

**Figure 2:**
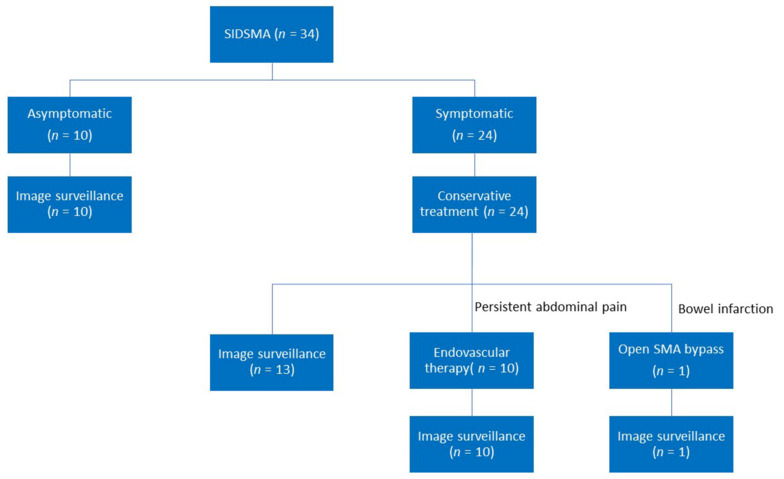
Patient treatment algorithm. SIDSMA: spontaneous isolated dissection of superior mesenteric artery, SMA: superior mesenteric artery.

The asymptomatic SIDSMA patients were detected incidentally during the abdominal workup. They all received conservative treatment with the preexisting medications and imaging surveillance at outpatient department. The symptomatic group was hospitalized in all cases, and they all received initial conservative treatment, which included food withdrawal, hydration, analgesia, and blood pressure control with the anti-impulse therapy first. Pain resolution was recognized as no more analgesics before or after oral intake. In selecting the antithrombotic agent, the presence of cardiovascular disease and the use of any antiplatelet agent before the onset of SIDSMA were considered. No additional antithrombotic agents were required if there were no pre-existing comorbidities. If a patient presented with suspicious bowel ischemia symptoms, persisted abdominal pain for more than two days, or progressive SMA dissection on CTA, catheter-based SMA stenting was performed with the 0.018 wire system via either the brachial or femoral approach with the self-expandable bare metal stents. The distal stent landing zone was chosen based on the angiography and was usually one centimeter beyond the distal end of SMA dissection. The size of the SMA stent was chosen based on the total diameter of dissected SMA distally and proximally. The size of the stent was usually 5 mm in the distal end, and telescoped to 8 mm proximally. The length of the stent was measured from the end of distal SMA dissection and extended 3–5 mm beyond the orifice of the SMA into the aorta. No distal intimal tear was ever noted. The antithrombotic agents were added with mono or dual antiplatelet agents. Open surgery was considered in patients after failed endovascular intervention and bowel infarction (Figure 2).

### 2.3. SMA Remodeling and Outcome Measurement

Positive SMA remodeling was defined as the FL reduction, TL expansion with no growth, or reduction in total SMA diameter during the follow-up. Once patients discharged after conservative or intervention treatment, they were periodically followed-up at an outpatient clinic to assess the recurrence of abdominal symptoms and morphologic changes of the SMA lesion at 3- and 6-month intervals and annually. SMA aneurysm was defined as more than 2.0 cm in diameter [14]. If no symptom recurrence or interval changes on CT images occurred after 1 year, CTA was performed biannually. In this study, SMA morphological comparison was only performed between EVT and conservative treatment.

### 2.4. Statistical Analysis

Binary variables were recorded as a number and percentage. Continuous variables were assessed as mean ± standard deviation or median with interquartile range as appropriate, and analysis was completed with student *t*-test and Mann–Whitney test. Potential risk factors were examined by simple and multiple linear regression with beta coefficient presented. The change in TL and FL diameters before and after intervention were graphed and analyzed by paired *t*-test and Wilcoxon Signed-rank test. Statistics were performed with SAS 9.4 software (SAS Inc., Cary, NC, USA), and *p*-value < 0.05 was considered statistically significant.

## 3. Results

During the study period from January 2007 to August 2019, a total of 34 consecutive patients with SIDSMA were identified (median age: 58 years, range: 47.3–64.8; male, *n* = 31; 91.2%: symptomatic, *n* = 24; 70.6%) at a single institution.

### 3.1. Patient Characteristics

Clinical characteristics are summarized in Table 1. Hypertension (*n* = 17, 50.0%) was the most common risk factor. Abdominal pain (*n* = 24, 70.6 %) was the most prominent symptom in SIDSMA and accounted for all symptomatic patients. In these 24 symptomatic patients, the median abdominal pain severity score was 6.0, and all these patients received initial conservative treatment. Twenty-three (67.6%) patients underwent primary conservative, and 11 (33.4%) patients underwent interventional treatments, respectively. The median time of abdominal pain was significantly longer in the interventional group compared to the medical treatment group. The median length of SMA dissection was 65.8 mm, range: 38.3–107.3. The patients were classified into four types according to Yun’s classification [6]: type I (*n* = 4, 11.8%), IIa (*n* = 5, 14.7%), IIb (*n* = 23, 67.6%), and III (*n* = 2, 5.9%). No significant differences were observed between the angiographic types regarding pain severity and duration at initial presentation. Clinical characteristics categorized by symptomatic or asymptomatic SIDSMA are illustrated in Supplementary Table S1.

### 3.2. Patients Outcomes

**Figure 3:**
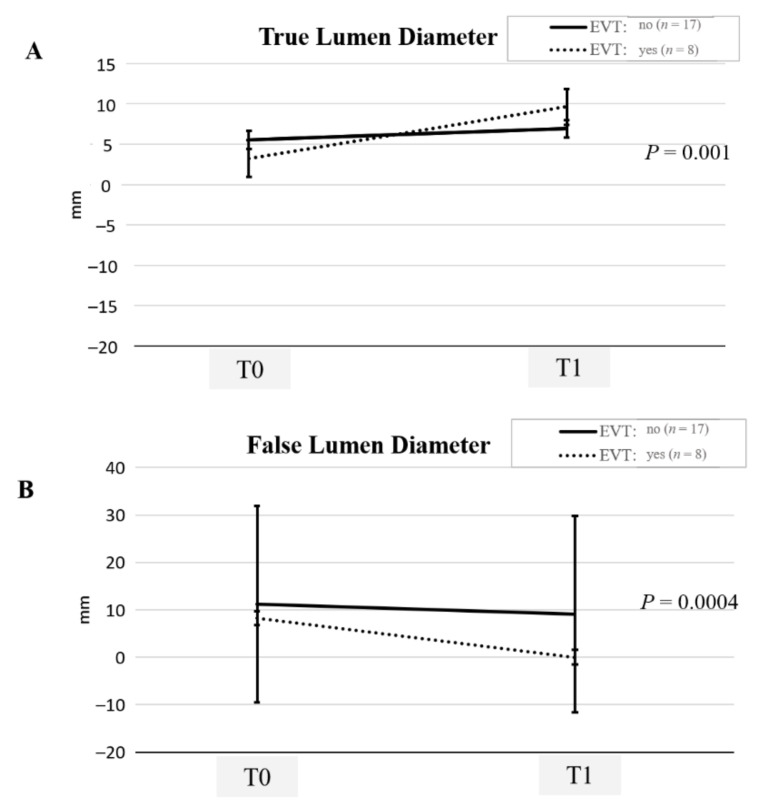
Superior mesenteric artery remodeling. (**A**) showing true lumen (TL) expansion and (**B**) false lumen (FL) reduction during the computed tomography scan follow-up. The TL expansion and FL reduction were significant better in patients undergoing superior mesenteric artery endovascular stenting (EVT).

In the interventional treatment, one patient required open laparotomy for SMA bypass with saphenous venous graft and bowel resection due to the mesenteric ischemia. This patient survived to discharge without sequelae. Ten (29.4%) patients were sent to the hybrid room (Artis zeego system, Siemens Healthcare, Forchheim, Germany) for EVT. There were no peri-procedural complications. All patients experienced symptoms relief after the intervention. Clinical features and morphologic changes on CTA were available in 25 (73.5%) patients during the mean follow-up of 23.3 months (range, 9.6–55.3 months). SMA remodeling could be observed in both conservative and EVT during the CTA follow-up at a median of 21.9 months, range:10.4–41.7. Compared to the conservative group, patients undergoing EVT had better SMA remodeling (Figure 3 and Supplementary Figure S1). After excluding asymptomatic cases, the SMA remodeling was still better in patients undergoing EVT (Supplementary Figure S2). In univariate study, SMA remodeling was significantly better in longer duration of abdominal pain, nausea, vomiting, interventional treatment, initial TL diameter, and length of SMA dissection. In the clinical outcomes, the initial presentation of abdominal pain, duration of symptom, EVT, and the length of SMA dissection were significantly related to duration of hospital stay and symptom relief. The use of anticoagulation and pain score were significantly related to the duration of symptom relief and hospital stay, respectively. However, EVT was the only significant risk factor for SMA remodeling (Table 2).

Comparing to conservative treatment, patients with Yun IIb classification were associated with better SMA remodeling after EVT (Table 3). Antithrombotic agents were not beneficial in either clinical outcomes or morphologic SMA remodeling.

At a median follow-up of 23.3 months (range: 9.6–55.2), no patients developed SMA aneurysm formation or recurrent symptoms requiring admission in these two groups. There was no stent occlusion or new dissection. The long-term survival was 96%. One patient died after 4 years follow-up in conservative treatment group unrelated to the SMA rupture or mesenteric ischemia.

## 4. Discussion

This study showed that SMA stenting in patients with SIDSMA was significantly better for SMA remodeling compared to medical treatment, especially in patients presenting with Yuan’s type IIb. After a median 23.3 months follow-up, no SMA aneurysm or deaths related to mesenteric ischemia were noted after either conservative and endovascular treatment.

SISMAD represented the dominant types of visceral artery dissection [11,14], and the incidence was reported to be approximately 0.06–0.08% [1,9,11]. In the current guideline, treatment of SIDSMA is aimed at controlling symptoms and preventing complications (e.g., bowel necrosis, aneurysm rupture) [9]. Most studies suggested initial treatment according to the clinical presentation on admission. If SIDSMA was identified as an incidental finding on CTA performed for other conditions, the patient could be managed by careful observation and conservative management [18,19]. None of the asymptomatic patients treated conservatively required secondary intervention [11]. However, in symptomatic patients, the failure rate ranged from 5.0–44.0% depending on the different reports [14]. In our study, 45.8% of symptomatic patients (11/24) had persistent abdominal pain and received the interventional therapy. One reason for this higher rate of intervention might be the threshold of the duration for conservative treatment. Currently there is no clear definition of the duration for the symptom relief indicating the failure of conservative treatment. The duration could range from a few days to weeks depending on the existing, or development of, collaterals [1,9,16]. Usually, conservative treatment would take longer to achieve symptom relief compared to surgery or EVT [16]. In our study, the average waiting period was two days after the initial abdominal pain to guide the interventional treatment option. The concern for bowel ischemia on clinical or radiologic examination was considered an indication for early intervention. Pain from the dissection itself was often confused with the presence of bowel ischemia and made the clinical evaluation difficult. Other laboratory markers such as leukocytosis, lactate, and amylase were neither sensitive nor specific. If any secondary signs of intestinal mal-perfusion, including bowel-wall thickening, pneumatosis, or lack of mucosal enhancement, appeared in the follow-up CTA, usually the golden period for the EVT had elapsed. The addition of antithrombotic agents did not prevent the progression of dissection and facilitate the development of collateral vessels in a short period of time [1,9,11,15,16,19], as shown in this study, where the antithrombotic agents did not prevent patients undergoing intervention treatment from receiving conservative treatment.

Given the weakening of the dissected SMA arterial wall, one might expect aneurysmal degeneration or recurrent dissection in the long-term follow-up, and the patients with favorable SMA remodeling overtime could be a surrogate for the long-term remission of the SIDSMA [9]. Currently, there is a uniform definition but only qualitative description regarding the SMA remodeling. Most studies compared the SMA remodeling as either complete or incomplete classifications. Complete remodeling was defined as SMA remodeling with no evidence of abnormality, and incomplete remodeling as increase in TL size with or without reduction in FL size in the follow-up CTA [15,16,17]. In the literature, the reported complete or incomplete remodeling rate after conservative treatment was quite variable; reported as 63.0% of patients at a mean follow-up of 16 ± 16 months after initial pain relief in the study from Heo et al. [16] and 12.6% in the study from Qiu et al. [17]. However, the reported rate of recurrent abdominal pain was quite similar in both studies (20.0–25.0%) [1,5,15,16,17]. Based on the reporting standard for type B aortic dissections, positive aortic remodeling should be classified as TL expansion or FL reduction with or without reduction of total aortic diameter [20]. In this study, we adopted the principle to define the SMA remodeling based on the TL, FL, and total arterial diameter change and compared between the EVT and conservative treatment. In our study, patients undergoing EVT achieved significantly better SMA remodeling (quantitative measurement, instead of qualitative description only) compared to patients receiving conservative treatment. In our study, we could not appreciate the importance of SMA remodeling in the recurrent abdominal pain because no recurrent symptoms were noted in both groups. It seemed that the recurrent abdominal pain was not really related to the SMA remodeling but more related to the collateral formation from the celiac or IMA axis after the dissection event.

Regarding the morphologic difference, symptomatic patients usually had longer length of SMA dissection than asymptomatic patients did [11].The average length of dissection ranged from 6.5 cm to 9.5 cm [15,17]. In our study, the median length of SMA dissection was 65.8 mm, and a significant difference was found between interventional and conservative treatment groups. More than six morphological classifications of SIDSMA have been proposed [1,5,9,16,21]. All were based on imaging appearance of the SIDSMA, such as the extent of the FL, entry and/or reentry visible, and presence of thrombosis in the FL and/or TL. However, none of these classifications could predict the clinical course. In our study, patients with Yun’s IIb classification CT morphology had significant better SMA remodeling in CTA follow-up in the treatment group, but no difference in midterm outcomes compared to patients receiving the medical treatment. This represented that patient who were symptomatic with dismal morphological appearance could have same midterm outcome after EVT treatment.

In the literature, the range of hospital stay was around 7 days in the conservative treatment group. In our study, comparing to patients under conservative treatment, patients undergoing interventional therapy had longer hospital stay because of the prolonged initial conservative treatment and the post-procedure recovery stay. For those symptomatic patients who received conservative treatment initially, secondary intervention was required at an estimated 12.0% of the SIDSMA patients during the follow-up [11]. However, in our study, no readmission for secondary intervention was related to the adverse events from SMA, including recurrent abdominal pain, aneurysmal formation, or new dissection. There was only one case requiring open SMA bypass surgery with saphenous vein graft from right common iliac artery and segmental bowel resection in early 2007. No other open surgical case was performed afterward. It may have been due to early diagnosis by CTA and early intervention management for prolonged abdominal pain before bowel necrosis happened. There was no EVT failure requiring conversion to open surgery in this study. The long-term survival was 96% at the median follow-up of 23.3 months. One patient died after 4 years follow-up in conservative treatment group unrelated to the SMA rupture or mesenteric ischemia. The long-term survival was compatible to previous reports explaining that rarely was the cause of mortality related to SMA rupture or ischemia [5,15,16].

This study has some limitations. First, it is a retrospective study in a single institution with a limited number of patients due to the rarity of SIDSMA. The assessment of sample size from previous studies and statistical power was not available due to the variations of the treatment modality [16,17]. As a result of our small sample size, multivariable analysis was not possible in some variables. The result might not be generalizable. However, it still remained the first study in the literature with the quantitative measurement of the SMA remodeling. Second, several patients did not have follow-up CTA images available in our electronic record system, and the follow-up imaging and timing were not uniform. In the CT scan, we could not differentiate the quantitative collateral circulations between symptomatic and asymptomatic patients.

## 5. Conclusions

In this study, SIDSMA could be initially managed by medical treatment. In symptomatic SIDSMA, EVT could be performed if the symptom persisted and before the mesenteric ischemia progressed. SMA remodeling was significantly better after EVT, especially with Yun’s IIb classification morphology. Anticoagulation therapy had no impact on the natural history of the SIDSMA. Secondary intervention was rarely required after both conservative and interventional treatments.

## Figures and Tables

**Table 1:** Patient demographics, CT scan morphology, and follow-up data.

	Total		Conservative		EVT		*p*-Value
	*n* = 34		*n* = 23		*n* = 11		
Age	58.0	47.3–64.8	58.0	46.0–65.0	58.0	51.0–62.0	0.392
Male	31.0	91.2%	21.0	91.3%	10.0	90.9%	0.704
BMI	25.3	22.4–27.2	26.0	21.7–27.9	24.6	22.8–26.1	0.274
Smoking	9.0	26.5%	6.0	26.1%	3.0	27.3%	0.625
Alcohol	5.0	14.7%	3.0	13.0%	2.0	18.2%	0.529
Hypertension	17.0	50.0%	12.0	52.2%	5.0	45.5%	0.362
SBP (mm Hg)	143.0	135.0–164.0	143.0	137–164	141.0	135.0	0.202
DBP (mm Hg)	90.0	83.0–97.0	90.0	82–95	91.0	83.0	0.388
DM	4.0	11.8%	2.0	8.7%	2.0	18.2%	0.389
Dyslipidemia	3.0	8.8%	3.0	13.0%	0.0	0.0%	0.296
CAD	1.0	2.9%	0.0	0.0%	1.0	9.1%	0.324
Medications							
Beta-blocker	28	82.3%	19	82.6%	9	81.8%	0.478
ARB or ACEI	26	76.4%	17	73.9%	9	81.8%	0.312
Pain duration (day)	1.0	0.0–2.0	1.0	0.0–1.0	2.0	1.5–3.5	0.001
Pain score	6.0	0.0–7.0	6.0	0.0–7.0	5.0	4.5–7	0.197
Abdominal pain	24.0	70.6%	14.0	60.9%	10.0	90.9%	0.077
Nausea	6.0	17.6%	3.0	13.0%	3.0	27.3%	0.288
Vomiting	7.0	20.6%	4.0	17.4%	3.0	27.3%	0.404
CT morphology							
SMA dissection length (mm)	65.8	38.3–107.3	62.0	22.3–87.1	80.0	66.0–117.0	0.034
TLD_T0 (mm)	12.0	11.0–13.0	12.0	11.0–13.0	12.0	11.0–13.0	0.240
TD_T0 (mm)	5.0	4.0–6.0	6.0	5.0–6.0	4.0	2.0–5.0	0.005
FD_T0 (mm)	7.0	6.0–9.0	6.0	5.0–8.0	8.0	8.0–9.0	0.351
Yun classification							
I	4.0	11.8%	4.0	17.4%	0.0	0.0%	0.191
IIa	5.0	14.7%	4.0	17.4%	1.0	9.1%	0.471
IIb	23.0	67.6%	14.0	60.9%	9.0	81.8%	0.206
III	2.0	5.9%	1.0	4.3%	1.0	9.1%	0.549
ICU stay (day)	0.0	0.0–0.8	0.0–0.5	0.0–0.5	0.0	0.0–1.0	0.403
Hospitalization (day)	3.0	0.0–5.8	0.0–3.5	0.0–3.5	10.0	3.0–12.3	<0.001
Symptoms resolution (day)	1.0	0.0–2	0.0–2.0	0.0–2.0	1.0	1.0–7.5	0.013
Anticoagulation use	20.0	58.8%	11.0	47.8%	9	81.8%	0.063
							
Follow-up		*n* = 25		*n* = 17		*n* = 8	
		CT morphology					
TLD_T1 (mm)	11.0	9.4–13.0	12.0	10.8–13.0	9.5	8.0–10.2	0.189
TD_T1 (mm)	7.4	7.0–9.0	7.0	6.0–7.4	9.5	8.0–10.2	0.001
FD_T1 (mm)	1.8	0.0–5.8	5.3	1.4–6.8	0.0	0.0–0.0	0.119
Last CT scan (month)	21.9	10.4–41.7	31.4	12.8–42.4	16.4	4.7–25.6	0.162
Follow-up (month)	23.3	9.7–55.2	30.6	5.4–47.5	28.1	16.5–86.8	0.100

Data are presented as *n* (%) or mean ± standard deviation. Nonparametric continuous data are presented as median ± interquadrant range (25–75%). EVT: endovascular superior mesenteric artery stenting, BMI: body mass index, DM: diabetes mellitus, CAD: coronary artery disease, SBP: systolic blood pressure, DBP: diastolic blood pressure, ARB: angiotensin II receptor blocker, ACEI: angiotensin-converting enzyme inhibitor, CT: computed tomography, SMA: superior mesenteric artery, TLD_T0: initial total lumen diameter, TD_T0: initial true lumen diameter, FD_T0: initial false lumen diameter, TLD_T1: last total lumen diameter, TD_T1: last true lumen diameter, FD_T1: last false lumen diameter, ICU: intensive care unit.

**Table 2:** Univariate and multivariate analysis.

	TLD Difference (T1–T0)				TD Difference (T1–T0)				FD Difference (T1–T0)			
Univariate linear regression												
		95% CI				95% CI				95% CI		
	β	Lower	Upper	*p*-value	β	Lower	Upper	*p*-value	β	Lower	Upper	*p*-value
Age	−0.0002	−0.0088	0.0083	0.9587	−0.0036	−0.0163	0.0092	0.5682	0.0031	−0.0127	0.0188	0.6929
Gender	0.1509	−0.1180	0.4198	0.2576	−0.1191	−0.5300	0.2918	0.5547	0.2730	−0.2285	0.7745	0.2723
BMI	0.0184	−0.0064	0.0431	0.1382	−0.0081	−0.0468	0.0306	0.6695	0.0239	−0.0182	0.0659	0.2526
Smoking	0.0973	−0.0985	0.2931	0.3147	0.0245	−0.2750	0.3240	0.8670	0.0790	−0.2756	0.4336	0.6497
Alcohol	0.2582	0.0049	0.5115	0.0461	−0.0286	−0.4425	0.3853	0.8874	0.2809	−0.2199	0.7816	0.2584
Hypertension	−0.0711	−0.2484	0.1063	0.4155	−0.0629	−0.3309	0.2051	0.6320	0.0000	−0.3288	0.3288	1.0000
SBP (mm Hg)	−0.9559	−2.2559	0.3442	0.1440	−0.0206	−1.2024	1.1611	0.9718	−0.9352	−1.8844	0.0139	0.0532
DBP (mm Hg)	−0.6980	−2.122	0.726	0.3255	0.1984	−1.0839	1.4806	0.7547	−0.8964	−1.9310	0.1383	0.0872
DM	−0.4092	−0.8327	0.0144	0.0576	0.2133	−0.4672	0.8938	0.5231	−0.6256	−1.4387	0.1875	0.1254
Dyslipidemia	0.4263	0.1506	0.7020	0.0040	−0.2339	−0.7196	0.2517	0.3294	0.6538	0.1018	1.2057	0.0222
CAD	0.1117	−0.3446	0.5680	0.6175	0.5258	−0.1223	1.1740	0.1068	−0.4176	−1.2541	0.4189	0.3131
Medications												
Beta-blocker	−0.2517	−0.6937	0.1904	0.2548	0.1700	−0.2288	0.5688	0.3917	−0.4217	−0.7476	−0.0958	0.0128
ARB or ACEI	−0.4784	−0.7250	−0.2319	0.0004	0.0847	−0.1403	0.3098	0.4487	−0.5632	−0.7401	−0.3862	0.0000
Pain duration (day)	−0.0221	−0.0652	0.0210	0.2994	0.1065	0.0590	0.1539	0.0001	−0.1287	−0.1904	−0.0670	0.0002
Pain score	−0.0008	−0.0248	0.0232	0.9447	0.0262	−0.0079	0.0602	0.1254	−0.0262	−0.0693	0.0169	0.2216
Abdominal pain	−0.0008	−0.1882	0.1865	0.9927	0.3293	0.0876	0.5710	0.0097	−0.3171	−0.6358	0.0015	0.0510
Nausea	−0.1160	−0.3351	0.1031	0.2849	0.4060	0.1188	0.6932	0.0076	−0.5219	−0.8763	−0.1675	0.0057
Vomiting	−0.0782	−0.2861	0.1296	0.4440	0.3133	0.0287	0.5979	0.0324	−0.3920	−0.7455	−0.0385	0.0312
CT morphology												
SMA dissection length	−0.0105	−0.0318	0.0109	0.3226	0.0319	0.0022	0.0616	0.0362	−0.0377	−0.0735	−0.0020	0.0395
TLD_T0 (mm)	0.0084	−0.0434	0.0601	0.7405	−0.0252	−0.1021	0.0516	0.5039	0.0330	−0.0626	0.1285	0.4836
TD_T0 (mm)	0.0091	−0.4806	0.4988	0.9697	−1.3132	−1.7782	−0.8481	<.0001	1.2188	0.5233	1.9143	0.0014
FD_T0 (mm)	0.0085	−0.0439	0.0609	0.7406	−0.0108	−0.0892	0.0677	0.7793	0.0181	−0.0791	0.1153	0.7045
Yun I	0.0000	.	.	.	0.0000	.	.	.	0.0000	.	.	.
Yun IIa	−0.0467	−0.4167	0.3234	0.7957	0.0600	−0.4547	0.5747	0.8108	−0.1067	−0.7532	0.5398	0.7355
Yun IIb	−0.1404	−0.4242	0.1434	0.3153	0.3137	−0.0810	0.7085	0.1133	−0.4433	−0.9371	0.0505	0.0760
Yun III	0.0067	−0.4070	0.4204	0.9736	0.5167	−0.0588	1.0921	0.0759	−0.5100	−1.2328	0.2128	0.1575
Treatment												
Anticoagulation	−0.0555	−0.2339	0.1229	0.5261	0.1142	−0.1506	0.3790	0.3817	−0.1565	−0.4796	0.1665	0.3273
EVT	−0.1126	−0.2991	0.0740	0.2244	0.5034	0.3135	0.6933	<0.0001	−0.6107	−0.8570	−0.3644	<0.0001

Multiple linear regression												
EVT	−0.0746	−0.2376	0.0884	0.3520	0.2042	0.0149	0.3935	0.0362	−0.3006	−0.5819	−0.0192	0.0377
	Duration of symptoms						Hospital stay					
Univaraite linear regression												
			95% CI					95% CI				
	β		Lower	Upper	*p*-value		β	Lower		Upper		*p*-value
Age	−0.0541		−0.1711	0.0629	0.3533		−0.0650	−0.2436		0.1137		0.4643
Gender	−0.8065		−5.6300	4.0171	0.7357		−0.5806	−7.9174		6.7561		0.8729
BMI	−0.1145		−0.3519	0.1230	0.3326		−0.0884	−0.5190		0.3423		0.6780
Smoking	0.0933		−3.0132	3.1999	0.9516		1.1733	−3.5265		5.8732		0.6146
Alcohol	1.8000		−2.0153	5.6153	0.3438		0.6207	−5.2532		6.4945		0.8309
Hypertension	−2.1765		−4.8033	0.4504	0.1012		−2.8235	−6.8611		1.2141		0.1640
SBP(mm Hg)	2.6422		−6.9878	12.2722	0.5801		7.1445	−7.4514		21.7404		0.3262
DBP(mm Hg)	1.7217		−8.7277	12.1710	0.7394		4.3084	−11.5657		20.1824		0.5842
DM	3.6667		−0.3773	7.7107	0.0740		4.8500	−1.3710		11.0710		0.1221
Dyslipidemia	−0.6559		−5.4824	4.1706	0.7837		−1.9785	−9.2835		5.3266		0.5850
CAD	−0.2727		−8.3843	7.8389	0.9458		−2.5455	−14.8328		9.7419		0.6759
Medications												
Beta-blocker	2.3333		−0.9293	5.5960	0.1549		3.6667	−1.2788		8.6121		0.1408
ARB or ACEI	1.6842		−0.1221	3.4905	0.0666		4.0000	1.2268		6.7732		0.0061
Pain duration (day)	0.8062		0.1241	1.4883	0.0220		1.3816	0.3715		2.3916		0.0089
Pain score	0.3061		−0.0631	0.6753	0.1010		0.5685	0.0202		1.1168		0.0426
Abdominal pain	3.2083		0.4309	5.9857	0.0249		5.7667	1.6969		9.8365		0.0069
Nausea	0.2857		−3.3082	3.8796	0.8724		2.8690	−2.4933		8.2314		0.2839
Vomiting	1.2857		−2.0723	4.6437	0.4412		3.1852	−1.8340		8.2044		0.2054
CT morphology												
SMA dissection length	0.4811		0.0421	0.9201	0.0327		0.4627	0.0457		0.8797		0.0306
TLD_T0 (mm)	−0.2562		−1.1679	0.6554	0.5710		−0.5638	−1.9407		0.8130		0.4104
TD_T0 (mm)	−8.0605		−14.3173	−1.8036	0.0132		−19.0484	−26.9669		−11.1299		<0.0001
FD_T0 (mm)	−0.1159		−1.0388	0.8070	0.7998		−0.2314	−1.6321		1.1693		0.7387
Yun I	0.0000		.	.	.		0.0000	.		.		.
Yun IIa	0.6000		−4.6048	5.8048	0.8155		1.7500	−6.0641		9.5641		0.6507
Yun IIb	3.1739		−1.0294	7.3772	0.1335		5.0978	−1.2126		11.4083		0.1094
Yun III	0.5000		−6.2194	7.2194	0.8802		8.7500	−1.3379		18.8379		0.0867
Treatment												
Anticoagulation	2.7571		0.1552	5.3591	0.0385		3.3500	−0.7044		7.4044		0.1021
EVT	3.1028		0.3944	5.8112	0.0261		7.5020	3.9657		11.0383		0.0001
Multiple linear regression												
EVT	0.7793		−2.3222	3.8807	0.6099		2.9964	−0.1124		6.1052		0.0582

β: unadjusted regression coefficient, T0: initial computed tomography diameter, T1: last computed tomography diameter measurement, BMI: body mass index, DM: diabetes mellitus, CAD: coronary artery disease, d: day, SBP: systolic blood pressure, DBP: diastolic blood pressure, ARB: angiotensin II receptor blocker, ACEI: angiotensin-converting enzyme inhibitor, CT: computed tomography, SMA: superior mesenteric artery, TLD_T0: initial total lumen diameter, TD_T0: initial true lumen diameter, FD_T0: initial false lumen diameter, EVT: endovascular superior mesenteric artery stenting.

**Table 3:** SMA remodeling stratified by CT morphology and EVT.

		Conservative				EVT				
SMA Remodeling	CT Morphology	*N* = 17	Median	IQR	(Q25–Q75)	*N* = 8	Median	IQR	(Q25–Q75)	*p*-Value
TLD difference (T1–T0)	Yun I	3	0.000	0.220	(−0.120–0.100)	0	NA	NA	NA	
	Yun IIa	3	−0.020	0.260	(−0.200–0.060)	0	NA	NA	NA	
	Yun IIb	10	−0.055	0.400	(−0.400–0.000)	7	−0.170	0.300	(−0.400–0.100)	0.3671
	Yun III	1	0.000	0.000	(0.000–0.000)	1	NA	NA	NA	
TD difference (T1–T0)	Yun I	3	0.000	0.100	(0.000–0.100)	0	NA	NA	NA	
	Yun IIa	3	0.100	0.180	(0.000–0.180)	0	NA	NA	NA	
	Yun IIb	10	0.140	0.150	(0.100–0.250)	7	0.700	0.400	(0.400–0.800)	0.0266
	Yun III	1	0.100	0.000	(0.100–0.100)	1	NA	NA	NA	
FD difference (T1–T0)	Yun I	3	−0.100	0.220	(−0.120–0.100)	0	NA	NA	NA	
	Yun IIa	3	−0.200	0.360	(−0.300–0.060)	0	NA	NA	NA	
	Yun IIb	10	−0.300	0.700	(−0.560–0.140)	7	−0.800	0.100	(−0.800–0.700)	0.0120
	Yun III	1	−0.100	0.000	(−0.100–0.100)	1	−1.000	0.000	(−1.000–1.000)	

Data are presented as presented as median ± interquartile range (IQR) (25–75%). T0: initial computed tomography diameter, T1: last computed tomography diameter measurement, TLD: initial total lumen diameter, TD: initial true lumen diameter, FD: initial false lumen diameter, EVT: endovascular superior mesenteric artery stenting, SMA: superior mesenteric artery, CT: computed tomography.

## Data Availability

Not applicable.

## Supplementary Materials

The following supporting information can be downloaded at: https://www.mdpi.com/article/10.3390/jcm11020465/s1, Figure S1: Total superior mesenteric artery diameter change during the computed tomography scan follow up; Figure S2: Total, true and false lumen of superior mesenteric artery diameter change during the computed tomography scan follow up after excluding asymptomatic cases; Table S1: Patients demographic, CT scan morphology and follow-up data.
