# Supplementary material for: Comparison of Superior Mesenteric Artery Remodeling and Clinical Outcomes between Conservative or Endovascular Treatment in Spontaneous Isolated Superior Mesenteric Artery Dissection
Source: J Clin Med. 2022 Jan 17;11(2):465. doi: 10.3390/jcm11020465 (PMC8777763; doi:10.3390/jcm11020465)
Supplement: Supplementary file 1 [file jcm-11-00465-s001.zip › jcm-1478754 suppl Table S1.pdf]

Journal of
Clinical Medicine

Table S1. Patients demographic, CT scan morphology and follow-up data.

	Total		Asymptomatic SIDSMA		Symptomatic SIDSMA		*p* Value
	n =34		n=10		n=24		
Age	58.0	47.3–64.8	63.0	59.8–65.0	55.0	46.5–61	0.033
Male	31.0	91.2%	9.0	90.0%	22.0	91.7%	0.440
BMI	25.3	22.4–27.2	23.8	22.2–25.2	26.2	22.4–27.3	0.093
Smoking	9.0	26.5%	1.0	10.0%	8.0	33.3%	0.085
Alchohol	5.0	14.7%	0.0	0.0%	5.0	20.8%	0.063
Hypertension	17.0	50.0%	5.0	50.0%	12.0	50.0%	0.500
SBP(mm-Hg)	143.0	135.0–164.0	135.0	116.0–140.0	147.0	137.0–166.0	0.010
DBP(mm-Hg)	90.0	83.0–97.0	80.0	68.0–90.0	92.5	83.0–97.3	0.027
DM	4.0	11.8%	0.0	0.0%	4.0	16.7%	0.090
Dyslipidemia	3.0	8.8%	1.0	10.0%	2.0	8.3%	0.440
CAD	1.0	2.9%	0.0	0.0%	1.0	4.2%	0.263
Medications							
Beta-blokcer	28	82.3%	8	80.0%	21	87.5%	0.484
ARB or ACEI	26	76.4%	7	70.0%	18	75.0%	0.339
Pain duration (day)	1.0	0.0–2.0	0.0	0.0–0.0	1.0	1.0–3.0	0.000
Pain score	6.0	0.0–7.0	0.0	0.0–0.0	7.0	5.8–8.0	<0.001
EVT	11.0	32.4%	1.0	10.0%	10.0	41.7%	0.038
Nausea	6.0	17.6%	0.0	0.0%	6.0	25.0%	0.043
Vomiting	7.0	20.6%	0.0	0.0%	7.0	29.2%	0.029
CT morphology							
SMA dissection length (mm)	65.8	38.3–107.3	41.5	20.5–62.0	75.0	60.9–121.0	0.009
TLD_T0 (mm)	12.0	11.0–13.0	12.0	11.5–12.8	12.0	10.9–13.0	0.068
TD_T0 (mm)	5.0	4.0–6.0	5.9	5.1–6.0	5.0	3.0–6.0	0.146
FD_T0 (mm)	7.0	6.0–9.0	6.5	5.8–7.0	8.0	5.5–9.0	0.087
Yun classification							
I	4.0	11.8%	3.0	30.0%	1.0	4.2%	0.203
IIa	5.0	14.7%	2.0	20.0%	3.0	12.5%	0.388
IIb	23.0	67.6%	4.0	40.0%	19.0	79.2%	0.245
III	2.0	5.9%	1.0	10.0%	1.0	4.2%	0.453
ICU stay (day)	0.0	0.0–0.8	0.0	0.0–0.0	0.0	0.0–2.0	0.021
Hospitalization (day)	3.0	0.0–5.8	0.0–3.5	0.0–3.5	10.0	3.0–12.3	<0.001
Symptoms resolution (day)	1.0	0.0–2.0	0.0	0.0–0.0	2.0	1.0–3.5	0.012
Anticoagulation use	20.0	58.8%	2.0	20.0%	18.0	75.0%	0.001
Follow up		n=25		n=17		n=8	
CT morphology							
TLD_T1 (mm)	11.0	9.4–13.0	11.0	9.4–12.5	11.0	9.8–13.0	0.104
TD_T1 (mm)	7.4	7.0–9.0	7.0	6.0–7.4	8.0	1.0–10.0	0.021
FD_T1 (mm)	1.8	0.0–5.8	5.2	2.6–6.0	0.0	0.0–5.5	0.062
Last CT scan (month)	21.9	10.4–41.7	41.7	10.3–44.2	19.5	11.0–33.5	0.190
Follow up (month)	23.3	9.7–55.2	44.2	8.1–68.6	25.9	11.0–49.6	0.401

Data are presented as n (%) or mean ± stand deviation. *Non-parametric continuous data are presented as median ± interquadrant range (25%-75%). SIDSMA: spontaenous isolated dissection of superior mesenteric artery, EVT: endovascular superior mesenteric artery stenting, BMI: body mass index, DM: diabetes mellitus, CAD: coronary artery disease, SBP: systoloc blood pressure, DBP: diastolic blood pressure, ARB: angiotension II receptor blocker, ACEI: angiotension converting enzyme inhibitor, CT: computed tomography, SMA: superior mensenteric artery, TLD_T0: initial total lumen diameter, TD_T0: initial true lumen diameter, FD_T0: initial false lumen diameter,TLD_T1: last total lumen diameter, TD_T1: last true lumen diameter, FD_T1: last false lumen diameter, ICU: intensive care unit.
