# Supplementary material for: Comparison of Superior Mesenteric Artery Remodeling and Clinical Outcomes between Conservative or Endovascular Treatment in Spontaneous Isolated Superior Mesenteric Artery Dissection
Source: J Clin Med. 2022 Jan 17;11(2):465. doi: 10.3390/jcm11020465 (PMC8777763; doi:10.3390/jcm11020465)
Supplement: Supplementary file 1 [file jcm-11-00465-s001.zip › Suppl Figure S1.pdf]

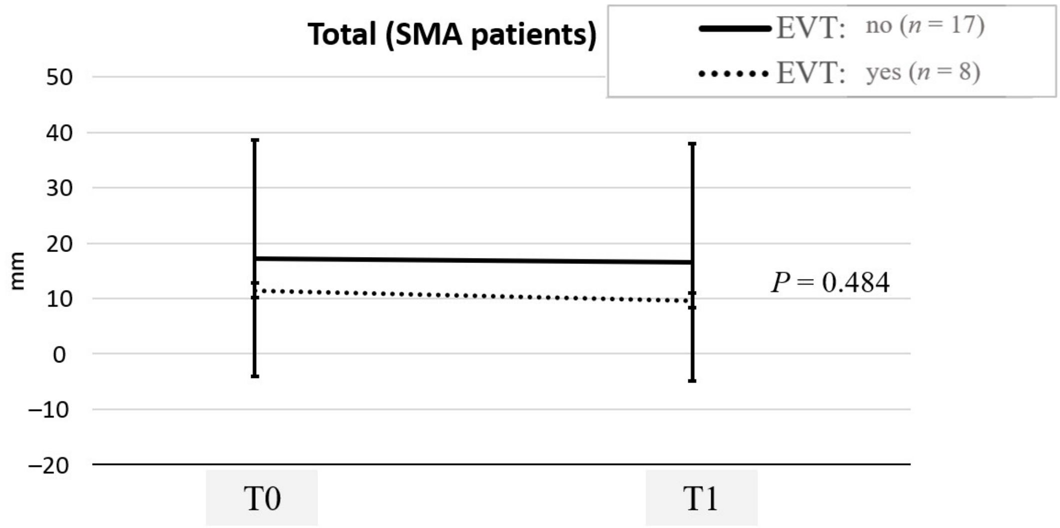

Supplementary Figure S1. Total superior mesenteric artery diameter change during the computed tomography scan follow up. No significant change was noted between the conservative and endovascular treatment group.
